# Health outcomes, education, healthcare delivery and quality – 3050. A survey of severe allergic symptoms among children in a disaster-hit area: A challenge of Tohoku Medical Megabank Organization

**DOI:** 10.1186/1939-4551-6-S1-P219

**Published:** 2013-04-23

**Authors:** Mami Ishikuro, Sahiro Kikuya, Riko Kuriyama, Chizuru Yamanaka, Taku Obara, Hirohito Metoki, Atsushi Hozawa, Ichiro Tsuji, Shinichi Kuriyama

**Affiliations:** 1 Tohoku University Tohoku Medical Megabank Organization, Japan; 2 Allergy Pot: Network Supporting Children with Allergy Certified Non-Profit Organization, Japan

## Introduction

On March 11, 2011, the Tohoku region and some prefectures along the Pacific coast of Japan were hit by the Great East Japan Earthquake. Past experiences of tsunami in other countries also raised concern about the health of the local people after the disaster. To monitor the health status of people living in Miyagi prefecture and to ensure sufficient quality of healthcare for them, Tohoku University has established the Tohoku Medical Megabank Organization (ToMMo). We plan to conduct three cohort studies of schoolchildren, three-generation families and adults. Here, we report the cohort study of schoolchildren. The aim of the schoolchildren cohort study is to grasp the current status of children’s health after the disaster, focusing on allergic diseases, neuropsychiatric disorders and so on as the main outcome measures. Here we introduce the methodology of our research and its progress, focusing on allergic diseases.

## Methods

The target population comprises children attending primary or secondary school in Miyagi prefecture, and we intend to conduct a screening survey for two years. We will attempt to confirm whether the prevalence of bronchial asthma and atopic dermatitis has been high, using the criteria of the International Study of Asthma and Allergies in Childhood (ISAAC) for bronchial asthma and atopic dermatitis. We will also confirm whether patients have been able to obtain appropriate treatment based on the guidelines for the treatment and management of bronchial asthma stipulated by the Japanese Society of Pediatric Allergy and Clinical Immunology. During the screening survey, we will first distribute questionnaires to the parents through the schools. In addition, we will ask about treatment status if children are found to have either of these diseases. Once completed, the questionnaires will be returned to the ToMMo directly by mail. Subsequent intervention will take place after the screening survey. This October, we will start a pilot study in three city and towns, where there are ten primary schools, five secondary schools and one welfare school, including 4,000 children as potential subjects. During the presentation we will report the rate of response to the questionnaire and some of the results obtained.

